# Cyclodextrin-Based Supramolecular Complexes of Osteoinductive Agents for Dental Tissue Regeneration

**DOI:** 10.3390/pharmaceutics13020136

**Published:** 2021-01-21

**Authors:** Masahiko Terauchi, Atsushi Tamura, Yoshinori Arisaka, Hiroki Masuda, Tetsuya Yoda, Nobuhiko Yui

**Affiliations:** 1 Department of Maxillofacial Surgery, Graduate School of Medical and Dental Science, Tokyo Medical and Dental University (TMDU), 1-5-45 Yushima, Bunkyo, Tokyo 113-8549, Japan; terauchi.org@tmd.ac.jp (M.T.); masumfs@tmd.ac.jp (H.M.); yoda.mfs@tmd.ac.jp (T.Y.); 2 Department of Organic Biomaterials, Institute of Biomaterials and Bioengineering, Tokyo Medical and Dental University (TMDU), 2-3-10 Kanda-Surugadai, Chiyoda, Tokyo 101-0062, Japan; arisaka.org@tmd.ac.jp (Y.A.); yui.org@tmd.ac.jp (N.Y.)

**Keywords:** regenerative medicine, biomaterials, cyclodextrin, inclusion complex, polyrotaxane

## Abstract

Oral tissue regeneration has received growing attention for improving the quality of life of patients. Regeneration of oral tissues such as alveolar bone and widely defected bone has been extensively investigated, including regenerative treatment of oral tissues using therapeutic cells and growth factors. Additionally, small-molecule drugs that promote bone formation have been identified and tested as new regenerative treatment. However, treatments need to progress to realize successful regeneration of oral functions. In this review, we describe recent progress in development of regenerative treatment of oral tissues. In particular, we focus on cyclodextrin (CD)-based pharmaceutics and polyelectrolyte complexation of growth factors to enhance their solubility, stability, and bioactivity. CDs can encapsulate hydrophobic small-molecule drugs into their cavities, resulting in inclusion complexes. The inclusion complexation of osteoinductive small-molecule drugs improves solubility of the drugs in aqueous solutions and increases in vitro osteogenic differentiation efficiency. Additionally, various anionic polymers such as heparin and its mimetic polymers have been developed to improve stability and bioactivity of growth factors. These polymers protect growth factors from deactivation and degradation by complex formation through electrostatic interaction, leading to potentiation of bone formation ability. These approaches using an inclusion complex and polyelectrolyte complexes have great potential in the regeneration of oral tissues.

## 1. Introduction

The oral cavity plays a pivotal role in the maintenance of life through ingestion, chewing, and swallowing of food, as well as speech and articulation. Therefore, dental care is crucial in protecting oral functions to maintain the quality of life [1]. To this end, therapeutic strategies for maintaining the functions of oral tissues are important. Dental pharmacology differs from medical pharmacology in terms of handling drugs related to clinical dentistry [2]. For example, medications used for endodontic treatment, periodontal treatment, and oral mucosal diseases are not usually handled in the medical science field. In addition, dental diseases occur mainly in calcified tissues, such as teeth, alveolar bone, and jawbone. Therefore, developing drugs for hard tissues, such as preventive agents for dental caries, drugs that promote bone formation, and drugs that suppress bone resorption, is important. Repairing the loss of dental tissues using various biomaterials or medicines has long been a major challenge in dentistry. Regenerative treatment of tissues and organs using therapeutic proteins and cells has received considerable attention in recent years [3,4]. In dentistry, regenerative medicines for dental tissues, such as dental pulp, dentin, periodontal tissue, alveolar bone, jawbone, and oral mucosa have been widely conducted [5,6]. Although alveolar bone regeneration and periodontal disease treatments have been established, the reconstruction of widely defective bone remains a challenge in the field of maxillofacial and plastic surgery. Therefore, research on regenerative treatment for dental tissues needs to progress further to realize successful regeneration of oral functions.

Regenerative medicines based on therapeutic cells (e.g., stem cells) and signaling proteins (e.g., growth factors) have received a lot of attention as potential ways of regenerating defective tissues [7]. Additionally, drugs that stimulate tissue regeneration have also been considered as potential candidates for dental tissue regeneration. However, these therapeutic cells and molecules sometimes show insufficient regenerative effects owing to insufficient engraftment, deactivation, degradation, rapid elimination from the site of injection, and excretion from the body. To solve these problems, the use of scaffolds and drug delivery systems (DDS) to prolong or promote the efficacy of therapeutic cells and molecules have been postulated in the field of dental tissue regeneration [8,9,10]. Some clinically approved therapeutic proteins are encapsulated in the biodegradable polymer matrix. These scaffolds and drug carriers can encapsulate therapeutic molecules to maintain their biological activities, while releasing encapsulated payloads through diffusion, matrix degradation, and other mechanisms [11,12]. These biomaterial-based approaches are crucial for successful treatment. In general, biodegradable polymers such as collagen, gelatin, and biodegradable synthetic polymers are utilized. Collagen, the main component of extracellular matrix, is a widely utilized scaffolds because of its excellent biocompatibility, degradation under physiological conditions, and preferential interaction with cells and tissues [13]. Similarly, poly(lactic acid) (PLA) and poly(lactic-co-glycolic acid) (PLGA) are representative biodegradable synthetic polymers used as scaffold [14]. Because PLA and PLGA have superior biocompatibility and biodegradability, they have been widely used in various clinical treatments. Likewise, numerous polymeric scaffolds have been developed as scaffolds for therapeutic molecules.

In this review, we summarize the general approaches for the regeneration of dental tissues using therapeutic molecules. Additionally, we describe recent approach for enhancing the bioactivity of therapeutic molecules through the formation of an inclusion complex with cyclodextrins (CDs) and complexation with CD-based supramolecular polymers.

## 2. Regenerative Treatment and Drugs

### 2.1. Regenerative Treatment Using Cells and Signaling Proteins

Therapeutic cells, including somatic stem cells have become an important source for tissue repair through the secretion of regenerative molecules such as growth factors, cytokines, and extracellular matrix [11,15,16,17]. Because these therapeutic cells have great potential in the regeneration of defective tissue, cell-based therapy has become a major approach for regenerative treatment [18]. The implantation of therapeutic cells has also been investigated for the regeneration of oral tissue. For example, clinical trials for the implantation of autologous periodontal ligament-derived cell sheets have been conducted for periodontal regeneration [19]. Cell-based regenerative therapy is a promising therapeutic approach in the regeneration of oral tissues. However, it is costly and involves complicated regulation.

As an alternative to cell-based therapy, signaling proteins, including recombinant growth factors and cytokines have received considerable attention in the regeneration of defective tissues [20]. Various signaling molecules have been tested to date for the regeneration of bone tissues such as the alveolar bone. Bone morphogenetic protein (BMP) is a major growth factor that promotes bone formation. BMP was first discovered in 1965 [21], and a total of 20 BMPs have been identified so far [22]. Among them, six types of BMPs, including BMP-2 through BMP-7, belong to the transforming growth factor-β (TGF-β) superfamily due to their high homology with TGF-β [23]. Of these, BMP-2 has long been a focus of attention because of its strong ability to induce bone differentiation in vitro and bone regeneration in vivo [24,25,26,27]. However, BMP-2 is unstable and readily deactivated under physiological conditions. Additionally, high doses of BMP-2 are required to maintain the long-term activity of BMP-2 in bone regeneration. However, it has been reported that high doses of BMP-2 induce various side effects such as inflammation and bone resorption [28,29,30]. For this reason, biodegradable scaffolds, such as collagen sponges [13,31], gelatin hydrogels [32], and poly(lactic acid) [33], have been used to protect BMP-2 from deactivation and degradation, while providing controlled release of BMP-2 under physiological conditions through the degradation of the scaffolds. Indeed, a clinically approved BMP-2 formulation (Infuse Bone Graft) comprising recombinant human BMP-2 encapsulated in the absorbable collagen sponge has been used in the surgical treatment of spine and orthopedic trauma [34]. In addition, the BMP-2 formulation has been shown to be effective in oral maxillofacial fields such as sinus augmentation and alveolar ridge augmentation [35,36,37].

Fibroblast growth factor (FGF), which was discovered in pituitary extracts in 1973, has also received attention for its role in tissue regeneration and a total of 22 members of the FGF family have been identified so far [38]. All FGFs are multifunctional proteins with a wide variety of therapeutic and regenerative effects on various cells [39,40,41,42,43]. FGF-2 (basic FGF) has been used extensively for tissue regeneration because it stimulates angiogenesis by promoting vascular endothelial growth factor (VEGF) secretion and has therefore been established as a potential therapeutic agent in various fields. In the regeneration of oral tissues, genetically modified recombinant FGF-2 has been approved for periodontal tissue regeneration in Japan (general name is Trafermin, and commercial names are Fiblast Spray and Regroth) [44]. Other growth factors, such as platelet-derived growth factor (PDGF), TGF-β, and VEGF have also been investigated for their potential to promote bone regeneration [45].

Enamel matrix protein derivative (Emdogain) is approved for the regeneration of the periodontal tissues such as alveolar bone and is widely employed in periodontal tissue regeneration [46]. The enamel matrix protein derivative is a mixture of various proteins, including amelogenins extracted from the enamel matrix. Amelogenins are active components of the enamel matrix derivative, which are involved in the formation of enamel and periodontal attachment during tooth development [47]. Therefore, the enamel matrix effectively regenerates periodontal tissue [48]. Emdogain Gel is an injectable gel formulation comprising a mixture of enamel matrix protein derivative and propylene glycol alginate (PGA). This formulation readily forms a hydrogel under physiological temperature and pH conditions. Gelation prevents the rapid excretion of therapeutic components at the site of injection and prolongs the therapeutic effects.

Recently, demineralized freeze-dried bone allograft, which is a bone graft material for promoting bone formation [49], has been used clinically to promote bone formation around the implant and to regenerate periodontal tissue. In the United States, materials obtained by demineralizing and freeze-drying human cadaver bone are sold as bone supplemental material.

### 2.2. Small-Molecule Osteoinductive Agents

**Figure 1:**
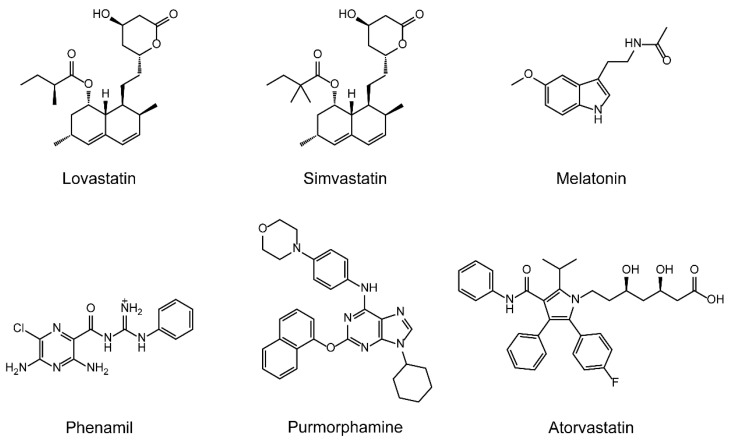
Chemical structure of small-molecule drugs that stimulate bone formation.

As mentioned above, various bone regeneration strategies have been developed, such as cell-based regenerative therapy and treatment with signaling proteins [50,51]. Apart from cell-based therapy and therapeutic proteins, small-molecule weight drugs that can stimulate bone formation have attracted attention because they are inexpensive and chemically stable compared with recombinant proteins (Figure 1). Munday et al. tested more than 30,000 reagents to identify a compound that specifically upregulated BMP-2 gene expression [52]. They found that lovastatin and simvastatin (SV), inhibitors of 3-hydroxy-3-methylglutaryl coenzyme A reductase [53,54], specifically upregulated BMP-2 gene expression. These statins are generally utilized in the treatment of hyperlipidemia through the reduction of plasma cholesterol and low-density lipoprotein levels. In addition, these statins have anti-inflammatory, anti-oxidant, and immunomodulatory properties, and their pleiotropic effect has attracted attention in various fields [55]. The discovery of the osteoinductive effects of statins is unexpected and interesting. SV is currently regarded as a major drug for stimulating bone regeneration, and the controlled release of SV from biodegradable scaffolds such as hydrogels, nanofibers, and microparticles has been demonstrated to potentiate bone regeneration [56,57]. SV has also been tested for the regeneration of alveolar bone [58].

Additionally, various drugs for stimulating bone regeneration have also been identified (Figure 1). Melatonin (MLT) is a hormone produced by the pineal gland. It plays a key role in the regulation of various physiological and pathophysiological processes, such as hypothalamic control of circadian rhythm, sexual activity, development, and immunomodulation [59]. MLT promotes osteogenic differentiation and mineralization in osteoblastic cells, and the mechanism and feasibility of using MLT as a potential bone regenerative agent have been investigated [59,60,61,62]. Other osteoinductive drugs, such as phenamil, purmorphamine, and some synthetic statins (e.g., atorvastatin) have also been shown to promote bone formation [63,64].

## 3. Inclusion Complexes of β-Cyclodextrin with Osteoinductive Drugs

### 3.1. Cyclodextrins and Inclusion Complexes

Cyclodextrin (CD) is a cyclic oligosaccharide comprising multiple α-D-glucopyranosides linked through α-1,4-linkages. CDs comprising 6, 7, and 8 glucose units are called α-, β-, and γ-CD, respectively. The internal diameter and depth of the cavity of these CDs increases with the increase in the number of glucose units. CDs are water-soluble, but the interior cavity is believed to be hydrophobic. Therefore, various hydrophobic small molecules are encapsulated in the cavity of CDs, and the complexes are called inclusion complexes (or host-guest complexes). The formation of inclusion complexes with CDs improves the physicochemical properties of guest molecules, such as solubility and chemical stability [65,66]. In general, CD can encapsulate various guest molecules at 1:1 or 2:1 (host:guest) stoichiometric molar ratios, depending on the size or polarity of guest molecules. Inclusion complexes containing CDs have been utilized in many industries including the food, cosmetic, and pharmaceutical industries.

**Figure 2:**
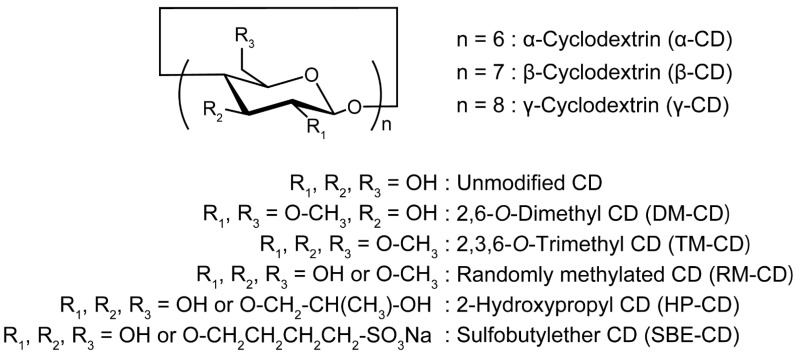
Chemical structures of cyclodextrins and representative chemically modified cyclodextrin derivatives.

The pharmaceutical applications of CDs have received considerable attention because CDs can be used as excipients to encapsulate various hydrophobic drugs into their cavities. The inclusion complex formation of hydrophobic drugs with CDs results in improved aqueous solubility, chemical stability, and bioavailability of drugs. Numerous studies have been conducted on the applications of CDs in the pharmaceutical field [65,66]. For the pharmaceutical application of CD, 2, 3, and 6 positions of hydroxy groups are usually modified with functional groups to increase their solubility in aqueous media and modulate the binding affinity to target guest molecules. In the case of β-CDs, chemically modified β-CD derivatives are employed because the aqueous solubility of unmodified β-CD is limited to 18.5 mg/mL, which is remarkably lower than the aqueous solubility of α-CD (145 mg/mL) and γ-CD (232 mg/mL) [66]. Although various chemically modified CD derivatives have been reported, the widely used modifications particularly for pharmaceuticals are methyl, 2-hydroxypropyl (HP), and sulfobutyl groups (Figure 2). These chemical modifications improve the solubility of β-CD by more than 500 mg/mL [66].

CD-based pharmaceutics are considered valuable in pharmacological approaches to oral diseases and bone regeneration. For example, Liu et al. reported an osteotropic alendronate-β-CD conjugate (ALN-β-CD) as a drug delivery carrier for targeting bone tissues and improving therapeutic effects in skeletal diseases [67]. The ALN-β-CD conjugate showed strong binding to hydroxyapatite, the main component of the skeleton. In a bilateral rat mandible model, the inclusion complex between ALN-β-CD and prostaglandin E1 stimulated a strong local bone anabolic reaction. Interestingly, ALN-β-CD itself could promote new bone formation at the site of injection.

### 3.2. Inclusion Complex of Simvastatin

As mentioned above, SV is a potential drug for promoting bone regeneration. However, SV is a hydrophobic molecule, and its apparent 1-octanol-water partition coefficient (log *P*_ow_) is 2.06 at pH 7 [68]. This indicates that the solubility of SV in aqueous media is poor. Although bone regenerating drugs are generally used during surgery, especially for oral tissues, the solubility of drugs needs to be improved for bone regenerative therapy. Note that SV formed an inclusion complex with β-CDs to improve the solubility in aqueous solutions [69,70,71,72,73]. However, the osteoinductive ability of the inclusion complexes containing SV is unclear. The osteoinductive abilities of HP-β-CD/SV and RM-β-CD/SV inclusion complexes in MC3T3-E1 cells were recently investigated [74]. Both HP-β-CD and RM-β-CD form an inclusion complex at a 1:1 molar ratio, and the HP-β-CD shows a higher apparent stability constant with SV (*K*_1:1_ = 1.24 × 10^3^ M^−1^) than it did with RM-β-CD (*K*_1:1_ = 1.01 × 10^3^ M^−1^).

**Figure 3:**
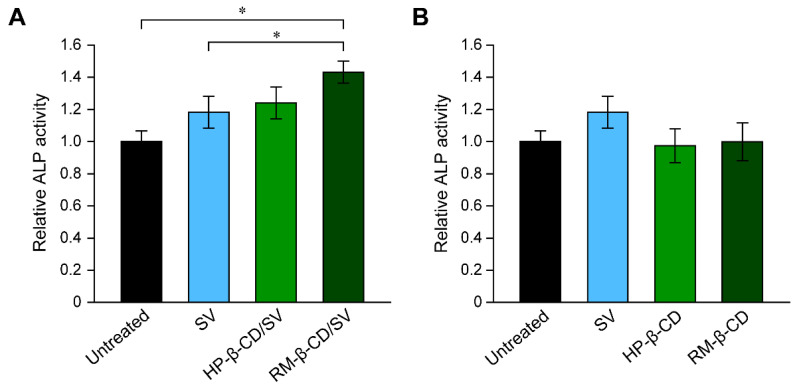
(**A**) Relative ALP activity in MC3T3-E1 cells after treatment with free SV (100 nM), HP-β-CD/SV inclusion complexes (SV: 100 nM, HP-β-CD: 1 μM), and RM-β-CD/SV inclusion complexes (SV: 100 nM, RM-β-CD: 1 μM) for 14 days. (**B**) Relative ALP activity in MC3T3-E1 cells after treatment with free SV (100 nM), HP-β-CD (1 μM), and RM-β-CD (1 μM) for 14 days. Data are expressed as the mean ± S.D. (*n* = 3, * *p* < 0.05). Reproduced with permission from [74] © 2016 Elsevier.

The osteoinductive abilities of the HP-β-CD/SV and RM-β-CD/SV complexes were evaluated in MC3T3-E1 cells. The treatment of MC3T3-E1 cells with SV promotes the production of osteogenic differentiation markers such as alkaline phosphatase (ALP), bone sialoprotein (BSP), and osteocalcin (OCN), and consequently leads to the formation of a mineralized matrix [75,76,77]. Treatment of MC3T3-E1 cells with free SV increased the production of ALP slightly at concentrations greater than 100 nM after 14 days (Figure 3A). It is interesting that the RM-β-CD/SV inclusion complexes containing 1 μM RM-β-CD and 100 nM SV significantly increased ALP production in MC3T3-E1 cells compared with that in the untreated and free SV-treated cells (Figure 3A). To confirm whether free HP-β-CD and RM-β-CD stimulated ALP production, MC3T3-E1 cells were treated with HP-β-CD and RM-β-CD for 14 days, and ALP production was evaluated in the same manner (Figure 3B). ALP production level of MC3T3-E1cells treated with free HP-β-CD and RM-β-CD was the same as that of the untreated cells. The mRNA expression of late-stage osteogenic marker proteins such as BSP and OCN was investigated using real-time RT-PCR to verify the effect of inclusion complexation of SV on the osteogenic differentiation of MC3T3-E1 cells. The mRNA expression of BSP and OCN significantly increased in the RM-β-CD/SV inclusion complexes group compared with that in the untreated and SV-treated groups [74]. Therefore, the SV-mediated osteogenic differentiation of MC3T3-E1 cells was significantly promoted by inclusion complexation with RM-β-CD, and the effect of free RM-β-CD on osteogenic differentiation was negligible.

Hydrophobic SV possesses good permeability and is easily taken into cells. The inclusion complexation between SV and β-CD derivatives improves solubility in water, but it leads to a reduction in membrane permeability because only the free form of the drug is capable of permeating lipophilic membranes [65,78]. Therefore, the inclusion complexes of SV with low stability constant might show higher uptake into cells and osteogenic differentiation activity. Consequently, inclusion complexation is a potential pharmaceutical approach for simultaneously improving the solubility and osteogenic differentiation activity of small-molecule osteoinductive drugs.

### 3.3. Inclusion Complexes of Other Osteoinductive Drugs

Similar to the inclusion complex of SV, the osteoinductive effects of other inclusion complexes containing MLT and phenamil have been demonstrated [79,80]. The preparation of inclusion complexes containing MLT has been reported using 2-hydroxypropyl β-CD (HP-β-CD) [81,82,83,84]. However, the effect of the inclusion complex of MLT with HP-β-CD on osteogenic differentiation is unknown. Recently, the osteogenic differentiation activity of HP-β-CD/MLT inclusion complexes in comparison to free MLT was investigated [79].

**Figure 4:**
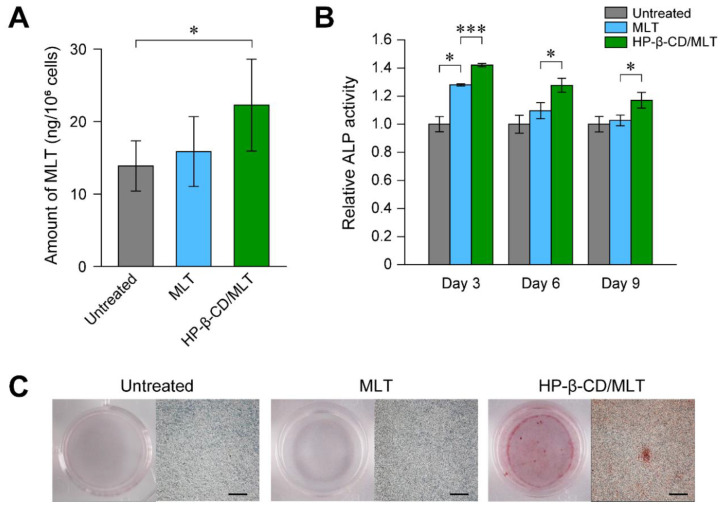
(**A**) Intracellular amount of melatonin (MLT) in MC3T3-E1 cells treated with free MLT (MLT: 1 μM) and HP-β-CD/MLT inclusion complexes (MLT: 1 μM, HP-β-CD: 1 μM) for 24 h. Data are expressed as the mean ± S.D. (*n* = 3, * *p* < 0.05). (**B**) Relative ALP activities in MC3T3-E1 cell treated with free MLT (100 nM) and HP-β-CD/MLT inclusion complexes (MLT: 100 nM, HP-β-CD: 100 nM) for 3, 6, and 9 days. Data are expressed as the mean ± S.D. (*n* = 3, * *p* < 0.05, *** *p* < 0.005). (**C**) Mineralized matrix formation of MC3T3-E1 cells as detected by staining with alizarin red. The cells were treated with free MLT (MLT: 100 nM) and HP-β-CD/MLT inclusion complexes (MLT: 100 nM, HP-β-CD: 100 nM) for 28 days (scale bars: 500 μm). Reproduced with permission from [79] © 2018 Elsevier.

HP-β-CD forms an inclusion complex with MLT at a 1:1 molar ratio with an apparent stability constant of *K*_1:1_ = 1.25 × 10^2^ M^−1^. The cellular uptake of MLT by MC3T3-E1 cells after 24 h treatment was studied using gas chromatography-mass spectrometry (Figure 4A). Although the treatment of MC3T3-E1 cells with MLT did not significantly increase the intracellular amounts of MLT, the inclusion complexation of MLT with HP-β-CD significantly increased the intracellular amounts of MLT compared with those of untreated cells. This result suggested that the inclusion complexation of MLT with HP-β-CD contributed to the increase in the cellular uptake efficiency of MLT.

To verify the osteogenic differentiation activity of the MLT inclusion complex, the relative ALP activity in MC3T3-E1 cells treated with free MLT (100 nM) and HP-β-CD/MLT inclusion complex (MLT: 100 nM, HP-β-CD: 100 nM) were investigated. After 3 days of treatment, MC3T3-E1 cells treated with free MLT showed higher ALP activity than the untreated cells (Figure 4B). However, the relative ALP activity in MLT-treated MC3T3-E1 cells decreased with increasing treatment time. In the case of MC3T3-E1 cells treated with HP-β-CD/MLT inclusion complex, the treated MC3T3-E1 cells showed significantly higher ALP activity than free MLT-treated cells at days 3, 6, and 9 of treatment. The mineralization of the treated MC3T3-E1 cells was assessed using alizarin red staining to further elucidate the enhanced osteogenic differentiation ability of HP-β-CD/MLT inclusion complex. The cells treated with HP-β-CD/MLT inclusion complexes exhibited approximately 4-fold greater deposition of mineralized matrix than the untreated cells after 28 days of treatment (Figure 4C). The inclusion complex of MLT promoted osteogenic differentiation efficiency in MC3T3-E1 cells, similar to the SV inclusion complex.

Jahed et al. reported on the use of inclusion complexes of phenamil, a small molecule that induces bone formation through upregulation of the TRB3 gene, to improve solubility [80]. To enhance the penetration of phenamil, a histidine-conjugated β-CD derivative (His-β-CD) was synthesized for intracellular delivery. Phenamil/His-β-CD complexes significantly promoted osteogenic differentiation compared with phenamil/HP-β-CD. Therefore, the chemical modification of β-CD plays an important role in enhancing the efficacy of osteoinductive drugs.

## 4. Enhanced Therapeutic Effects of Growth Factors Using Anionic Polymers

### 4.1. Heparin and Other Anionic Polymers

Various therapeutic and pharmacological molecules, such as growth factors, small interfering RNAs, and statins, have been shown to promote bone regeneration efficiency [85,86,87,88]. Among them, BMP-2 is the most widely investigated due to its strong osteoinductive activity. However, the stability and pharmacokinetics of BMP-2 need to be improved. One method involves the use of a scaffold and DDS as mentioned above. Another method to improve the stability and bioactivity of BMP-2 is by using anionic polymers, chemically-modified natural polymers, and synthetic anionic polymers. Because BMP-2, FGF-2, and other growth factors have a heparin-binding domain, which typically contains cationic lysine and arginine [89], anionic polymers can bind to these growth factors via electrostatic interactions and hydrogen bonding. Takeda et al. reported that heparin enhances the osteogenic differentiation activity of BMP-2 in cultured cells [90]. Heparin protects BMP-2 from deactivation and degradation, presumably through complexation with BMP-2 or binding to the heparin-binding domain in BMP-2 [90,91], because the isoelectric point of BMP-2 is 8.5 [92]. Additionally, heparin enhances the ectopic bone formation activity of BMP-2 in mice [91]. Therefore, heparin is currently used as a component of the scaffold to stably encapsulate growth factors [93,94,95,96], because there is no interaction between clinically utilized collagen sponges and growth factors. For example, heparin is conjugated to a matrix component or complexed with other components [93,94,95,96]. However, the clinical use of heparin for this purpose might be limited owing to its strong anti-coagulant effects [97,98].

For this reason, other sulfated, sulfonated, or anionic polymers, such as chemically-modified natural polymers, tissue-derived heparan sulfate, and synthetic anionic polymers, have been examined, either as alternatives to heparin or to further improve the effects of heparin [90,91,99,100,101,102,103,104,105,106,107]. Additionally, the design and synthesis of heparin-mimetic polymers containing sulfate, sulfonate, and carboxylate have long been a focus of attention [107]. To date, various anionic polysaccharides and synthetic polymers have been identified as promoting the bioactivity of BMP-2 and other growth factors. The major anionic polymers, including sulfated natural polymers and synthetic anionic polymers used to enhance the activity of growth factors are summarized in Table 1. Although the polyelectrolyte complexation ability of these sulfated natural polymers and anionic polymers with growth factors was not characterized in all studies, these anionic polymers are considered to potentially form a complex with growth factors, leading to modulation of the stability and bioactivity of growth factors.

### 4.2. Supramolecular Polyrotaxane for Polyelectrolyte Complexation with BMP-2

**Figure 5:**
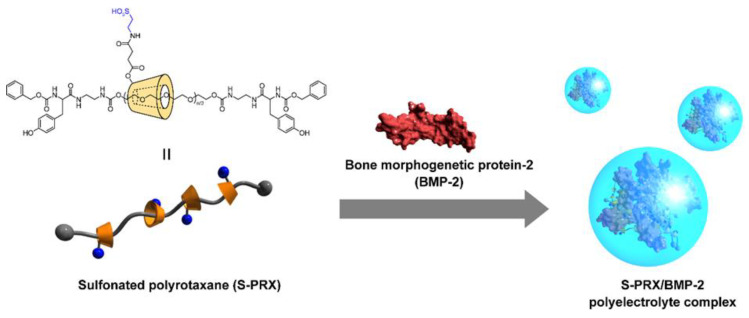
Chemical structure of sulfonated polyrotaxane (S-PRX), and the formation of a polyelectrolyte complex with BMP-2. Reproduced with permission from [127] © 2015 Wiley.

Polyrotaxanes (PRXs) are one of the representative supramolecular polymers comprising multiple cyclic molecules (e.g., α-CD) threaded onto a linear polymer axle (e.g., poly(ethylene glycol); PEG) capped with terminal bulky stopper molecules (Figure 5) [108,109,110,111]. The characteristic feature of PRXs is the molecular mobility of threaded α-CDs, and the threaded α-CDs in PRXs can be freely mobile along the polymer axle. However, PRXs show poor solubility in most solvents. Therefore, the chemical modification of hydroxy groups of threaded α-CDs with functional groups or hydrophilic ligands (e.g., peptides and saccharides) improves the solubility of PRXs in aqueous solutions [112,113,114,115,116]. This chemical modification enables the application of PRXs as biomaterials and drug carriers [117,118,119]. Interestingly, the molecular mobility in chemically modified PRXs contributes to improving steric hindrance in multivalent interactions or binding and complex formation with proteins. For example, the binding ability of maltose-conjugated PRXs to concanavalin A is greater than that of maltose-conjugated linear poly(acrylic acid). Because molecular mobility as predicted from the long spin-spin relaxation time of maltose moieties in PRXs is higher than those modified with conventional linear polymers, it is considered that the molecular mobility of threaded α-CDs facilitates binding with proteins [120]. Similarly, the association constant between mannose-conjugated PRXs and concanavalin A is approximately 200-fold higher than that of mannose-conjugated poly(acrylamide) [121]. Based on these results, the mobility of functional group-modified α-CDs in PRXs facilitates the interaction with proteins. Moreover, cationic or anionic functional group-modified PRXs can form polyelectrolyte complexes with oppositely charged proteins [122,123,124,125,126]. The movable features of α-CDs or rigid rod-like structures of PRXs can facilitate the formation of complexes with proteins and nucleic acids. Therefore, the supramolecular structure of PRXs might be a fascinating backbone for stable complex formation with BMP-2 compared with conventional linear polymers.

**Figure 6:**
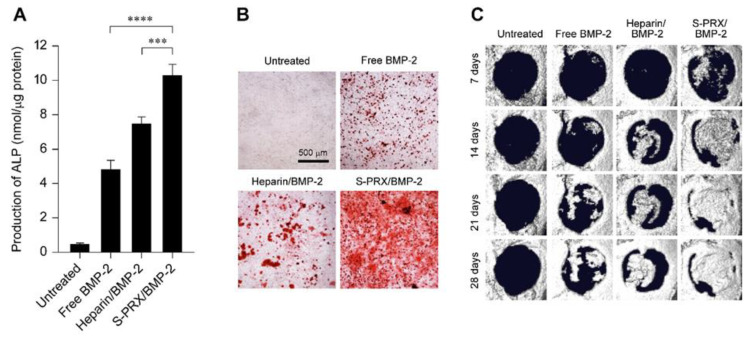
(**A**) Amount of ALP production in MC3T3-E1 cells after treatment with free BMP-2, heparin/BMP-2, and S-PRX/BMP-2 for 72 h. The concentrations of BMP-2 and the SPE-PRXs were 100 ng/mL and 1,000 μg/mL, respectively. The data are expressed as the mean ± S.D. (*n* = 3, *** *p* < 0.005, **** *p* < 0.001). (**B**) Alizarin red stained images for MC3T3-E1 cells after treatment with free BMP-2, heparin/BMP-2, and S-PRX/BMP-2 for 21 days. (**C**) X-ray μ-CT images of the mouse calvarial defect treated with free BMP-2, heparin/BMP-2, and S-PRX/BMP-2 complexes embedded into collagen sponges. The concentrations of BMP-2 and S-PRX were 100 ng/mouse and 1000 μg/mouse, respectively. The amount of implanted heparin was 100 μg/mouse. Reproduced with permission from [127] © 2015 Wiley and [128] © 2017 Wiley.

Based on this, a new modality for anionic polymers based on PRXs was designed for modulating the complexation with BMP-2 (Figure 5) [127,128]. Sulfonate groups are modified on the threaded α-CDs in PRXs, and the sulfonated PRX (S-PRX) has been tested to form complexes with BMP-2. Enzyme-linked immunosorbent assay (ELISA) and electron microscopic observation confirmed the formation of polyelectrolyte complexes between negatively charged S-PRX and positively charged BMP-2. The osteogenic differentiation ability of S-PRX/BMP-2 complexes (BMP-2: 100 ng/mL) in MC3T3-E1 cells was evaluated by measuring the secretion of ALP and comparing this with heparin/BMP-2 complex. The heparin/BMP-2 complexes showed higher ALP secretion than free BMP-2 (Figure 6A). Interestingly, the amount of ALP produced by treatment with S-PRX/BMP-2 complex was significantly higher than that of the treatment with heparin/BMP-2 complex. These results indicated that S-PRX has a superior ability to enhance BMP-2-induced osteogenic differentiation compared with heparin. Additionally, the mRNA expression levels of middle- to late-stage osteogenic differentiation markers, such as runt-related transcription factor 2 (Runx2), osterix, and OCN were evaluated. Cells treated with S-PRX/BMP-2 and heparin/BMP-2 complexes showed significantly higher mRNA expression of Runx2, osterix, and OCN than the free BMP-2-treated cells. To further investigate the osteogenic differentiation ability of S-PRX/BMP-2 complexes, the deposition of the mineralized matrix in MC3T3-E1 cells was demonstrated through alizarin red staining [129,130]. The untreated MC3T3-E1 cells showed negligible deposition of mineralized matrix even after 21 days (Figure 6B). In contrast, marked deposition of mineralized matrix was observed in MC3T3-E1 cells treated with free BMP-2 (100 ng/mL), heparin/BMP-2, and S-PRX/BMP-2 after 21 days of culture. Of these, the S-PRX/BMP-2-treated cells showed higher deposition of the mineralized matrix than the cells treated with free BMP-2 and heparin/BMP-2.

To clarify the bone regeneration ability of the S-PRX/BMP-2 complex [125], a mouse calvarial defect model which is widely utilized for evaluating the ability of bone regeneration, was employed [131]. The S-PRX/BMP-2 complexes were embedded in collagen sponges, which is the same as a clinically utilized BMP-2 formulation, and implanted in the region of the calvarial defect. In this experiment, the quantities of BMP-2 and S-PRXs implanted were 100 ng and 1000 μg, respectively. After the surgical implantation of the complexes, the area of newly formed bone was assessed using X-ray micro-computed tomography. Bone regeneration was not observed in any group 7 days post implantation, and the area of new bone was negligible (Figure 6C). However, new bone formation was observed in the BMP-2-implanted groups 14 days after implantation. Although the area of newly formed bone in mice implanted with free BMP-2 and heparin/BMP-2 complex increased after 21 and 28 days of implantation, the area of newly formed bone did not cover the entire area of the defect even 28 days post implantation. In sharp contrast, new bone formation in S-PRX/BMP-2 complex-implanted mice was remarkable and reached nearly complete bone regeneration at 28 days of implantation. These results confirmed that the S-PRXs exhibited more rapid and greater bone regeneration than any other treatment group.

Histological evaluation of the regenerated bone area confirmed the successful fusion of regenerated bone and original bone in mice implanted with the S-PRX/BMP-2 complex. In contrast, the fusion of regenerated bone and original bone was immature in mice implanted with the free BMP-2 and heparin/BMP-2 complex. Based on these results, S-PRX/BMP-2 complexes induced rapid and significant bone regeneration in comparison to widely utilized heparin, making S-PRX a promising candidate for enhancing BMP-2-mediated bone regeneration efficiency.

## 5. Conclusions

Alveolar bone regeneration and periodontal disease treatment have been established to improve the quality of life of patients. However, the reconstruction of widely defective bone to repair surgically removed and disease-related bone defects remains a challenge in the field of maxillofacial and plastic surgery. Therefore, research on regenerative treatments for dental tissues need to progress further to realize successful regeneration of oral functions. In this review, we described recent progress in the development of potential regenerative treatment for oral tissues. In particular, we focused on CD-based pharmaceutics and polyelectrolyte complexation of growth factors to enhance their bioactivity. Inclusion of osteoinductive small-molecule drugs by CDs improves solubility of the drugs in aqueous solutions and increases the in vitro osteogenic differentiation. The chemical modification of CD plays an important role in the complexation and osteogenic differentiation efficiency. To improve the bioactivity of growth factors, various anionic polymers such as heparin and its mimetic polymers have been developed. These polymers protect growth factors from deactivation and degradation, potentiating the activity of growth factors. Although these CD-based osteoinductive drugs and the complex of growth factors have a great potential to promote bone regeneration, the regeneration efficacy of oral tissues, including alveolar bone and widely defective bone, need to be clarified. As a future perspective, preclinical research and clinical trials of CD-based osteoinductive drugs and the complex of growth factors for the oral tissues are required for the development of novel dental regenerative treatments.

## Figures and Tables

**Table 1:** Anionic polymers to enhance the activity of growth factors.

Anionic Polymer	Growth Factor	Reference
Heparin	BMP-2	[90,91]
Dextran sulfate	BMP-2	[90]
Dermatan sulfate	BMP-2	[102]
2-*N*,6-*O*-sulfated chitosan	BMP-2	[103]
Cellulose sulfate	BMP-2	[104]
Chitosan sulfate	BMP-2	[104]
Poly(vinyl sulfonate)	FGF-2	[105]
Poly(glutamic acid)	BMP-2	[106]

## Data Availability

Not applicable.
